# The effect of project-based learning on students’ motivation in school-based physical Education and behavioral intention toward sport participation: a theoretical and empirical perspective in educational sport contexts

**DOI:** 10.3389/fpsyg.2026.1915768

**Published:** 2026-09-28

**Authors:** Lihong Liang, Yao Xia, Yu Huang

**Affiliations:** 1 Geely University of China, Chengdu, China; 2 Sichuan University, Chengdu, China

**Keywords:** intrinsic motivation, physical education, project-based learning, self-efficacy, sport engagement

## Abstract

This study examined the association of Project-Based Learning (PBL) with students’ motivation in physical education (PE) and behavioral intention toward sport participation within an integrated theoretical framework combining Self-Determination Theory (SDT), constructivist learning theory, and social cognitive theory. A quasi-experimental pretest–posttest control group design involved 120 secondary school students from eight intact classes, with four classes assigned to PBL (*n* = 60) and four to traditional instruction (*n* = 60). The 12-week intervention comprised structured sport-based PBL activities, whereas the control condition used predominantly teacher-centered instruction. Intervention effects were evaluated using linear mixed-effects models (LMM) accounting for repeated measurements and class-level clustering, followed by a baseline-adjusted two-level parallel mediation analysis accounting for students nested within classes. Significant group × time interactions favored PBL for overall motivation (*p* < 0.001, *d* = 1.60), intrinsic motivation (*d* = 1.67), identified regulation (*d* = 1.35), and behavioral intention (*d* = 1.48; all *p* < 0.001). Significant group × time interactions were also observed for psychological needs satisfaction, including autonomy (*d* = 1.72), competence (*d* = 1.80), and relatedness (*d* = 1.62), as well as self-efficacy (*d* = 1.70; all *p* < 0.001). Psychological needs satisfaction [*B* = 0.21, 95% CI (0.04, 0.38)] and self-efficacy [*B* = 0.16, 95% CI (0.02, 0.30)] showed significant indirect effects on post-intervention motivation, while the direct effect remained significant [*B* = 0.29, 95% CI (0.04, 0.54)], with a total effect of *B* = 0.66, 95% CI (0.37, 0.95), a pattern consistent with significant indirect associations. Because the mediators and outcome were assessed concurrently at posttest, these indirect effects should not be interpreted as definitive evidence of causal mediation. These findings indicate that PBL was associated with greater improvements in motivational and psychological outcomes and that psychological needs satisfaction and self-efficacy may represent complementary pathways linking PBL with students’ motivation toward PE and behavioral intentions toward continued sport participation.

## Introduction

Physical education (PE) in schools is increasingly positioned not only as a platform for physical development, but also as a strategic context for fostering lifelong engagement in physical activity and sport participation (Lee et al., 2021). Despite its recognized importance, many educational systems continue to face a persistent challenge: students’ declining motivation toward PE classes and school-based sports activities (Juwono and Szabo, 2020). This issue is particularly concerning in adolescence, a critical developmental stage in which motivational orientations toward physical activity are formed and consolidated. When students perceive PE as repetitive, skill-restrictive, or assessment-dominated, their intrinsic motivation tends to decrease, leading to reduced participation, lower engagement, and limited long-term physical activity adherence (Haug et al., 2023).

Contemporary motivational theories in sport and education, including Self-Determination Theory (SDT) and Achievement Goal Theory, emphasize that sustained participation in physical activity is strongly influenced by autonomy support, competence perception, and meaningful learning experiences (De Meester et al., 2016; Zhang and Ma, 2023). However, traditional PE instruction in many school contexts remains teacher-centered, technique-focused, and outcome-oriented, often limiting students’ active decision-making, creativity, and ownership of learning processes (López-Rodríguez et al., 2025). This instructional gap creates a mismatch between pedagogical delivery and the psychological needs required to sustain motivation in sport participation.

However, motivational and psychological determinants should be considered within a broader behavioral and socio-ecological context. Recent systematic review evidence indicates that adolescent interventions frequently target potentially modifiable individual and interpersonal determinants, including autonomous motivation, basic psychological needs, self-efficacy, behavioral intention, and peer-related factors, while evidence that changes in these determinants translate into objectively measured changes in physical activity or sedentary behavior remains limited (Ciaccioni et al., 2026; Kolovelonis et al., 2024). These findings suggest that autonomy, competence, relatedness, self-efficacy, and behavioral intention are important psychological determinants, but improvements in these constructs should not be regarded as equivalent to demonstrated or sustained changes in actual physical activity behavior.

In response to these challenges, Project-Based Learning (PBL) has emerged as a promising student-centered pedagogical approach that emphasizes inquiry, collaboration, problem-solving, and authentic learning experiences (Klopp and Stark, 2025; Wijnia et al., 2024). Within the context of education, PBL enables students to actively construct knowledge through real-world projects, encouraging deeper cognitive engagement and meaningful participation (Carcamo-oyarzun et al., 2020; Zhang and Ma, 2023). In PE settings, PBL can be operationalized through sport-related projects such as designing training programs, organizing mini-sport events, analyzing movement performance, or developing health-promotion campaigns (Ceppi-Larraína et al., 2021). These activities not only enhance physical competence but also promote autonomy, relatedness, and intrinsic interest in sport participation (Barnett et al., 2022). Although PBL has been widely studied in general education disciplines, its application within school-based PE and sport contexts remains relatively underexplored (Palmizal et al., 2026). Existing research has primarily focused on cognitive and academic outcomes such as critical thinking, collaboration skills, and subject comprehension, with limited attention to motivational mechanisms that drive sustained sport participation behavior (Draper et al., 2023).

More importantly, prior studies have rarely examined how PBL translates pedagogical experiences into sport-specific motivational outcomes such as students’ intention to participate in school-based physical activities and extracurricular sports programs (Sirwesvary and Jamaludin, 2025). In addition, the psychological mechanisms underlying this relationship—particularly the simultaneous role of basic psychological needs satisfaction (autonomy, competence, relatedness) and self-efficacy—remain insufficiently integrated in a single explanatory model within PE research (Cairney et al., 2012; den Uil et al., 2023).

This gap is important for two reasons. First, motivation in PE is not only a cognitive construct but also a behavioral driver that determines students’ actual participation in school sports activities. Second, understanding how innovative pedagogies such as PBL influence motivation requires an integrated theoretical and empirical approach that connects instructional design, psychological mechanisms, and observable behavioral outcomes (Barnett et al., 2016; Fernández-Espínola et al., 2022). Without such integration, the effectiveness of PBL in PE may remain conceptually promising but empirically under-validated.

Therefore, this study aims to examine the effect of PBL on students’ motivation toward school-based PE and sport participation from both theoretical and empirical perspectives (Carcamo-oyarzun et al., 2025). By integrating motivational theory with intervention-based evidence, this study seeks to clarify how PBL functions as a structured pedagogical strategy to enhance students’ intrinsic motivation, psychological need satisfaction, self-efficacy, and behavioral intention in PE settings (Carcamo-oyarzun et al., 2025; Carcamo-oyarzun and Estevan, 2020). Specifically, the study contributes to the literature by providing empirical evidence on motivational change induced by PBL and by positioning sport-based project learning as a theoretically grounded instructional model within school PE curricula.

From a theoretical standpoint, this research extends SDT by situating autonomy, competence, and relatedness within a project-based sport learning environment while simultaneously integrating social cognitive mechanisms to explain motivational regulation (Lee et al., 2021; Vasconcellos et al., 2020). From a practical perspective, the findings are expected to inform PE curriculum design by offering evidence-based guidance on how to restructure instructional approaches to enhance student engagement and sustained sport participation.

Importantly, this study advances the literature by proposing an integrated motivational model in which PBL operates not merely as a pedagogical strategy, but as a multidimensional intervention that simultaneously activates psychological need satisfaction and self-efficacy pathways, leading to sustained sport participation intention in school contexts.

In summary, this study addresses a critical gap in PE pedagogy by investigating whether and how PBL can serve as an effective instructional strategy to enhance students’ motivation toward school-based sport participation, thereby contributing to both educational innovation and long-term physical activity promotion.

### Theoretical framework

This study is grounded in an integrative theoretical framework that combines SDT, constructivist learning theory, and social cognitive theory to explain how pedagogical innovation in PE influences students’ motivation toward school-based sport participation. This integrative approach is necessary because motivation in PE is not a unidimensional construct, but rather the result of interacting psychological, pedagogical, and social processes that jointly determine students’ engagement, persistence, and behavioral intention in sport-related activities. However, existing literature has largely examined these theoretical perspectives in isolation, with limited integration between pedagogical design, psychological need satisfaction, and cognitive-behavioral mechanisms within PE contexts.

SDT provides the primary psychological foundation for understanding motivation in PE. SDT posits that the quality of motivation is determined by the satisfaction of three basic psychological needs: autonomy, competence, and relatedness. Autonomy refers to students’ perception of volition and psychological ownership in learning activities, competence refers to perceived effectiveness in performing physical and sport-related tasks, and relatedness refers to the sense of social connection with peers and teachers. When these needs are satisfied, students are more likely to internalize the value of physical activity, resulting in intrinsic motivation, sustained engagement, and persistent sport participation behaviors. Conversely, traditional teacher-centered PE characterized by repetitive drills, limited choice, and performance-oriented instruction may undermine autonomy support and weaken motivational quality, ultimately reducing long-term sport engagement.

Constructivist learning theory further strengthens this framework by emphasizing that learning is an active, contextualized, and socially mediated process in which students construct knowledge through experience, reflection, and interaction. In PE, learning is inherently experiential, as students develop motor skills, tactical understanding, and physical competence through direct engagement in movement-based tasks. From this perspective, effective pedagogical practice should provide authentic, meaningful, and problem-centered learning environments that allow students to actively explore, collaborate, and construct understanding. PBL aligns strongly with constructivist principles because it positions students as active agents in designing, implementing, and evaluating real-world sport-related projects. Through this process, students move beyond passive skill reproduction and instead engage in deep learning that integrates cognitive, physical, and social dimensions of sport participation.

In addition, social cognitive theory contributes a critical explanatory dimension by highlighting the dynamic interaction between personal factors, behavior, and environmental influences. A central construct within this theory is self-efficacy, defined as individuals’ belief in their capability to successfully perform specific tasks. In PE contexts, self-efficacy plays a decisive role in shaping motivation, persistence, and behavioral intention toward sport participation. PBL enhances self-efficacy through repeated mastery experiences, peer modeling, social comparison, and formative feedback embedded in collaborative sport-related tasks. Furthermore, the social structure of PBL fosters observational learning and reciprocal interaction among students, thereby strengthening motivational regulation and engagement in physical activity.

PBL itself serves as the central pedagogical mechanism integrating these theoretical perspectives. In this study, PBL is conceptualized not merely as a teaching strategy, but as a structured motivational learning system that activates psychological, cognitive, and social mechanisms simultaneously within PE environments. In practice, PBL transforms traditional instruction into a student-centered, inquiry-driven, and collaboratively structured learning process in which students engage in authentic sport-related projects such as designing fitness programs, organizing sport events, analyzing movement performance, and developing health promotion initiatives. These activities require students to make decisions, solve problems, and apply knowledge in meaningful contexts, thereby enhancing cognitive engagement and physical participation.

This pedagogical structure simultaneously supports autonomy through student agency in decision-making, enhances competence through progressive skill development and iterative feedback, and strengthens relatedness through sustained peer collaboration. In parallel, it reinforces self-efficacy by providing mastery experiences and social modeling opportunities that build confidence in physical performance. Importantly, this study proposes that these psychological and cognitive processes do not operate independently but interact synergistically to produce sustained motivational and behavioral outcomes in school-based sport participation.

Overall, the integration of SDT, constructivist learning theory, and social cognitive theory suggests that PBL influences students’ motivation in PE through a multi-layered mechanism involving psychological need satisfaction, active knowledge construction, and self-efficacy enhancement. As a result, PBL is theoretically expected to increase intrinsic motivation, strengthen behavioral intention toward sport participation, and promote sustained engagement in physical activity beyond the classroom setting. This integrated framework contributes a novel theoretical advancement by positioning PBL as a unified pedagogical-motivational system that bridges instructional design and motivational psychology to explain sport participation behavior in PE contexts.

### Conceptual model

Building upon the integrated theoretical framework of SDT, constructivist learning theory, and social cognitive theory, this study proposes a comprehensive conceptual model explaining how PBL influences students’ motivation toward PE and school-based sport participation. The model positions PBL as a pedagogical-motivational system that restructures the learning environment to simultaneously activate cognitive engagement, psychological need satisfaction, and behavioral intention toward sport participation.

In this model, PBL is conceptualized as the independent variable representing a student-centered, inquiry-driven pedagogical system characterized by authentic sport-related projects, collaborative problem-solving, reflective learning, and student agency in decision-making. Unlike traditional teacher-centered instruction, PBL reconfigures PE learning environments by embedding real-world sport tasks that require students to actively construct knowledge, interact socially, and apply physical skills in meaningful contexts. Importantly, PBL is not treated merely as an instructional strategy, but as a multi-layered learning system that simultaneously shapes cognitive, psychological, and behavioral dimensions of student engagement in PE.

The primary dependent variable in this study is students’ motivation toward PE and school-based sport participation, conceptualized as a multidimensional construct comprising intrinsic motivation, identified regulation, and behavioral intention toward sustained sport engagement. Motivation is considered a dynamic psychological state influenced by both internal psychological mechanisms and external pedagogical conditions, rather than a static or outcome-only variable.

The conceptual model proposes both direct and indirect effects of PBL on motivation. The direct pathway suggests that exposure to meaningful, authentic, and student-centered learning experiences may directly enhance students’ interest, enjoyment, and engagement in PE without requiring full mediation through psychological constructs. This reflects the assumption that experiential learning environments can immediately stimulate intrinsic motivational responses.

The indirect pathway operates through two interrelated psychological mechanisms derived from SDT and social cognitive theory. First, PBL enhances basic psychological needs satisfaction, including autonomy, competence, and relatedness. Autonomy is fostered through student decision-making in project design and execution, competence is developed through iterative mastery experiences in sport-related tasks, and relatedness is strengthened through sustained peer collaboration and shared project responsibility. These psychological needs function as key mediators that transform pedagogical experiences into internalized motivational regulation.

Second, the model incorporates self-efficacy as an additional mediating construct derived from social cognitive theory. PBL strengthens self-efficacy through mastery experiences, peer modeling, feedback cycles, and iterative performance improvement in authentic sport tasks. Increased self-efficacy enhances students’ confidence in their ability to perform physical activities, thereby reinforcing persistence, engagement, and intention toward sport participation. In this model, self-efficacy is conceptualized not only as an individual cognitive belief but also as a dynamically reinforced construct that interacts with psychological need satisfaction to shape motivational outcomes.

A key novelty of this conceptual model is the integration of psychological need satisfaction and self-efficacy as parallel and interacting mediators rather than isolated explanatory pathways, providing a more comprehensive explanation of how pedagogical design translates into sport participation behavior in PE contexts. Overall, the model suggests that PBL influences students’ motivation through a multi-level mechanism involving direct experiential effects, psychological need satisfaction, and self-efficacy enhancement. These pathways collectively contribute to increased intrinsic motivation, stronger behavioral intention, and sustained engagement in school-based sport participation.

### Research hypotheses

Based on the integrated theoretical framework and the proposed conceptual model, this study formulates a set of hypotheses that examine both the direct and indirect effects of PBL on students’ motivation toward PE and school-based sport participation. These hypotheses are grounded in SDT, constructivist learning theory, and social cognitive theory, which collectively suggest that motivational outcomes in PE are shaped by pedagogical design, psychological need satisfaction, and self-efficacy beliefs. The hypothesized relationships among these constructs are illustrated in Figure 1, which provides a visual representation of the proposed direct, indirect, and potential psychological pathway within the study model.

**Figure 1:**
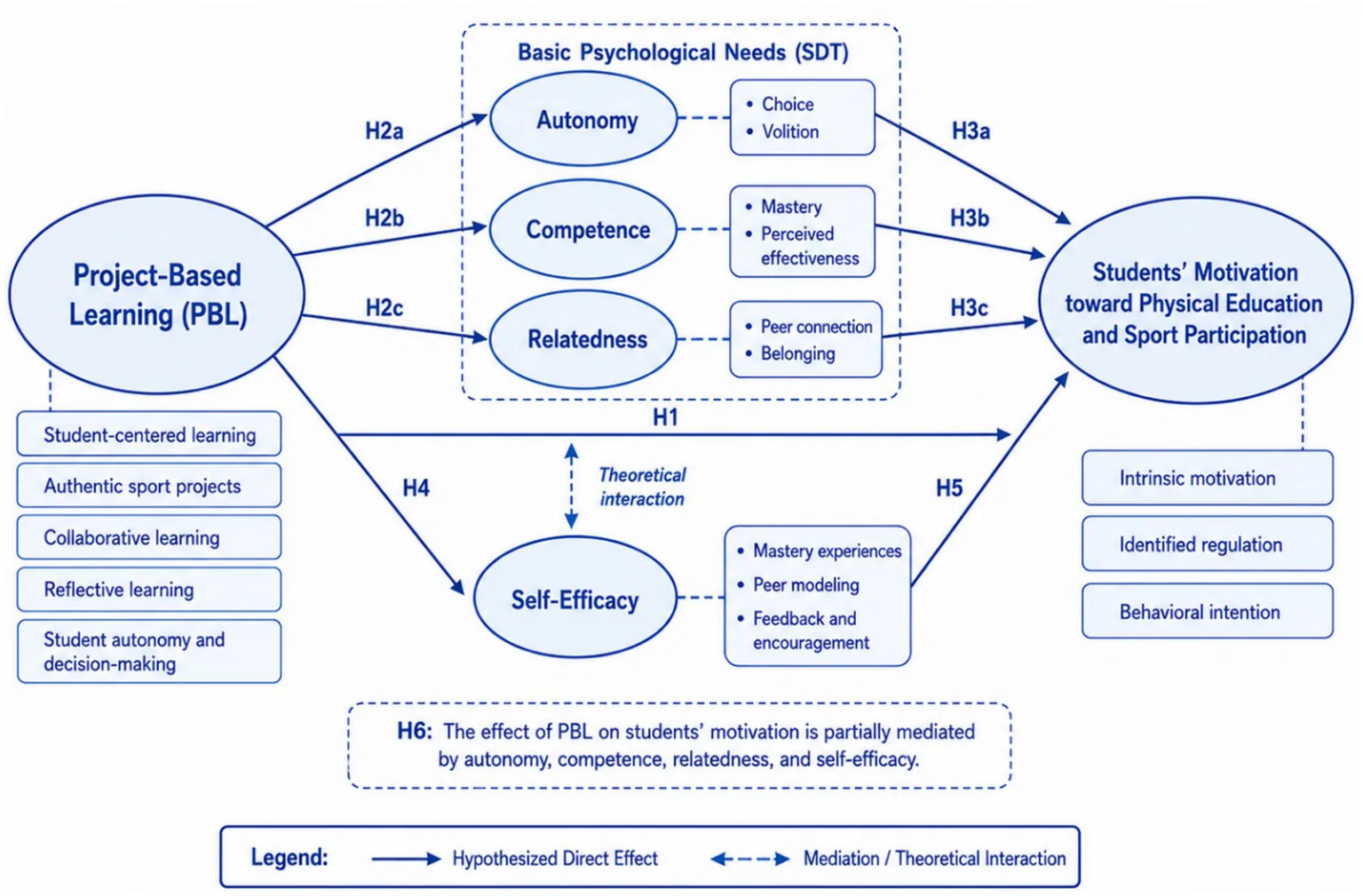
Hypothesized model illustrating the relationships among PBL, basic psychological needs, self-efficacy, and students’ motivation toward PE and sport participation. The model presents direct effects (H1, H4, H5), indirect effects via autonomy, competence, and relatedness (H2–H3), and a partial mediation mechanism involving psychological needs and self-efficacy (H6).

First, this study posits that PBL has a direct positive effect on students’ motivation in PE. Because PBL creates meaningful, student-centered, and experientially rich learning environments, it is expected to enhance students’ intrinsic interest, enjoyment, and behavioral intention toward sport participation. Therefore, it is hypothesized that PBL significantly increases students’ overall motivation toward PE and sport participation compared to traditional instructional approaches.

Second, based on SDT, it is hypothesized that PBL positively influences the satisfaction of basic psychological needs in PE contexts. Specifically, PBL is expected to enhance students’ perceived autonomy by providing opportunities for decision-making and self-directed learning in sport-related projects, improve perceived competence through progressive skill development and mastery experiences, and strengthen relatedness through collaborative group-based learning structures. Accordingly, PBL is hypothesized to significantly and positively affect autonomy, competence, and relatedness.

Third, this study hypothesizes that satisfaction of the three basic psychological needs (autonomy, competence, and relatedness) is positively associated with students’ motivation toward PE and sport participation. Students who experience higher levels of psychological need satisfaction are expected to demonstrate higher intrinsic motivation, stronger engagement in PE classes, and greater intention to participate in school-based sports activities.

Fourth, in line with social cognitive theory, this study hypothesizes that PBL positively enhances students’ self-efficacy in PE. Through repeated mastery experiences, peer modeling, feedback, and active engagement in authentic sport-related tasks, students are expected to develop stronger beliefs in their ability to successfully perform physical activities. Increased self-efficacy is, in turn, expected to contribute positively to motivational outcomes.

Fifth, it is hypothesized that self-efficacy significantly predicts students’ motivation toward PE and sport participation. Students with higher perceived self-efficacy are expected to demonstrate greater persistence, engagement, and willingness to participate in physical activity both inside and outside the classroom context.

Finally, this study proposes a mediation mechanism in which the effect of PBL on students’ motivation is partially mediated by psychological need satisfaction (autonomy, competence, and relatedness) and self-efficacy. This implies that PBL not only directly influences motivation but also exerts its effects indirectly through psychological and cognitive processes that are fundamental to sustained engagement in PE and sport participation. Collectively, these hypotheses provide a comprehensive and theoretically grounded structure for empirically examining the motivational impact of PBL in school PE settings. More importantly, they advance existing PE literature by proposing an integrated motivational mechanism in which pedagogical design (PBL), psychological need satisfaction, and cognitive self-efficacy jointly explain sport participation behavior, rather than treating these constructs as isolated or independent explanatory pathways as commonly seen in prior studies.

## Methodology

### Research design

This study employed a quasi-experimental pretest–posttest control group design to examine the effect of PBL on students’ motivation toward PE and school-based sport participation. This design was selected to ensure ecological validity within authentic school environments while enabling systematic comparison between an experimental group receiving PBL-based instruction and a control group receiving traditional teacher-centered instruction. The quasi-experimental design is particularly appropriate for educational settings where random assignment at the individual level is not feasible, while still allowing the examination of intervention effects on motivational outcomes under real classroom conditions. Compared to cross-sectional or correlational approaches commonly used in PE research, this design provides stronger empirical evidence for evaluating the effectiveness of pedagogical innovation over time. The study followed a structured pretest–posttest procedure, enabling both within-group and between-group comparisons to determine the effect of the intervention on students’ motivational development.

### Participants

Participants in this study were secondary school students enrolled in compulsory PE courses. The study involved a total of 120 students (*N* = 120) drawn from intact classes in participating schools, with 60 students assigned to the experimental group (PBL-based instruction) and 60 students assigned to the control group (traditional teacher-centered instruction). The study was conducted in two secondary schools and involved eight intact PE classes taught by four PE teachers. Each school contributed four participating classes, resulting in approximately 15 students per class. Group allocation was conducted at the class level to preserve natural classroom structures and to minimize contamination between instructional conditions. Consistent with the quasi-experimental design, the eight intact classes were allocated using a non-random class-level procedure, with four classes assigned to the PBL condition and four classes to the control condition; both instructional conditions were represented within each participating school. Each teacher taught two participating classes, with one class receiving PBL-based instruction and the other receiving traditional instruction, thereby reducing the potential confounding influence of teacher-specific characteristics. A cluster sampling technique using intact classes was applied to ensure ecological validity and to reflect authentic PE learning environments. This approach allowed the intervention to be implemented under real instructional conditions while maintaining methodological rigor in evaluating the effects of pedagogical innovation on students’ motivation. To further minimize teacher-related variation, all participating teachers followed standardized lesson objectives, session durations, assessment schedules, and instructional protocols, with the principal difference between conditions being the pedagogical approach used.

An *a priori* power analysis conducted using G*Power 3.1 indicated that approximately 128 participants were required to achieve 80% statistical power at a two-tailed alpha level of 0.05 for detecting a medium between-group effect (Cohen’s *d* = 0.50). The final sample comprised 120 students, with 60 participants in each group, representing a modest shortfall from the initially estimated target. Because G*Power does not explicitly account for cluster-based designs, this calculation was interpreted as an individual-level approximation. Given that students were nested within intact classes, the effective sample size may have been reduced by the design effect associated with within-class correlation. This shortfall was primarily attributable to the availability of intact classes and practical recruitment constraints within the participating school settings. To evaluate the statistical implications of the achieved sample size, an individual-level sensitivity analysis was conducted. With 60 participants per group, α = 0.05, and statistical power of 0.80, the achieved sample size corresponded to a minimum detectable between-group effect of approximately Cohen’s *d* = 0.52. Because this sensitivity analysis did not explicitly incorporate the class-level clustering of the study design, it was interpreted as an approximate indication of statistical sensitivity rather than as a formal power assessment of the mixed-effects model. Baseline characteristics were descriptively similar across the PBL and control conditions in terms of age, gender distribution, and prior exposure to PE content, suggesting broadly comparable initial profiles across groups. The participants were within the typical adolescent age range for secondary education, supporting developmental comparability across groups. These descriptive baseline similarities reduced concerns regarding major pre-intervention differences between the conditions, although they should not be interpreted as evidence of formal statistical equivalence. The study was conducted in a natural school setting to ensure high ecological validity and to capture authentic PE learning conditions, where outcomes are influenced by classroom dynamics, teacher interaction, and peer collaboration. Prior to participation, all students received full information regarding the study objectives, procedures, and voluntary participation. Informed consent was obtained through official school approval and parental or guardian consent procedures in accordance with ethical standards for research involving minors. All data were collected and processed anonymously to ensure confidentiality and academic research integrity.

### Intervention: project-based learning in physical education

The intervention was designed based on core principles of PBL, emphasizing student-centered inquiry, collaboration, and authentic sport-related problem-solving within PE settings. In the experimental condition, students engaged in structured sport-based projects embedded into the PE curriculum. These projects included designing fitness training programs, organizing mini sport events, analyzing movement performance, and developing health and physical activity promotion campaigns within the school environment. The PBL intervention followed a systematic instructional cycle consisting of four phases: (1) project initiation and problem identification, where students were introduced to real-world sport-related problems; (2) planning and collaboration, where students designed project strategies in groups; (3) project execution, involving active implementation of sport-related tasks; and (4) reflection and presentation, where students evaluated outcomes and shared their learning experiences. Throughout the intervention, teachers acted as facilitators by providing scaffolding, guidance, and feedback rather than direct instruction, thereby promoting student autonomy, engagement, and active learning.

The intervention was implemented over a period of 12 weeks (3 months), with two PE sessions per week, resulting in a total of 24 structured instructional sessions. This duration was selected to ensure sufficient exposure to the PBL model, allowing meaningful development of students’ motivational and psychological outcomes while maintaining feasibility within the school curriculum. The selected intervention length is consistent with prior PE intervention studies, which suggest that medium-term exposure is necessary to produce stable changes in motivation, self-efficacy, and learning engagement. Prior to implementation, the four participating PE teachers received standardized training covering the theoretical principles of PBL, the four-phase instructional cycle, teacher facilitation strategies, project organization, and the procedures required for both the PBL and control conditions. Standardized lesson guidelines, project instructions, learning objectives, and implementation checklists were provided to promote consistency across teachers and classes. Separate instructional protocols and lesson plans were provided for the PBL and control conditions to minimize cross-contamination between instructional approaches. Teachers were instructed to restrict project-based activities, student-led project planning, collaborative project tasks, and the four-phase PBL cycle to the experimental classes and not to introduce these elements into the control classes. Intervention fidelity was monitored using structured implementation checklists and periodic classroom observations focusing on adherence to the prescribed PBL phases, use of student-centered learning activities, collaborative project work, and the facilitator role of the teacher. Classroom observations also monitored adherence to the prescribed control-group instructional format and the absence of core PBL components in control lessons. Teachers also completed session logs documenting lesson completion, implementation of the planned activities, and student attendance. Any deviations from the assigned instructional protocol were documented in the session logs and reviewed during the intervention period. These records were reviewed throughout the 12-week intervention to monitor adherence to the intended instructional protocol. To minimize teacher-related variation, each participating teacher contributed to both instructional conditions, teaching one PBL class and one control class, while lesson duration, curriculum objectives, assessment schedules, and core PE content were standardized across conditions.

In contrast, the control group received traditional PE instruction characterized by teacher-centered approaches, including direct demonstration, repetitive skill drills, and structured physical activities with limited student autonomy and collaboration. Learning in the control condition primarily emphasized technical performance and teacher-directed practice rather than project-based engagement. To reduce spillover from the PBL condition, teachers were required to follow the designated control lesson plans and avoid introducing project-based tasks, student-led planning, or collaborative project activities that were specific to the PBL condition. Control classes followed the same intervention period, session frequency, curriculum objectives, and assessment schedule as the PBL classes, with the principal difference being the instructional approach. This procedural standardization was intended to reduce systematic differences attributable to exposure time, curriculum content, or teacher characteristics rather than to the pedagogical intervention itself. Overall, this structured implementation ensured methodological consistency, ecological validity, and internal validity, thereby strengthening the evaluation of PBL effects on students’ motivation toward PE and school-based sport participation.

### Control condition

The control group received conventional PE instruction that was predominantly teacher-centered and skill-oriented. Instruction consisted of direct demonstrations, repetitive technical drills, and standardized physical fitness activities. Learning activities were structured around teacher-led explanations and performance-based assessments, with limited opportunities for student decision-making, collaboration, or project-based engagement. The instructional content and learning objectives were aligned with the standard PE curriculum to ensure comparability with the experimental group. The control condition was delivered over the same duration and frequency as the intervention group (12 weeks, two sessions per week), ensuring equivalent instructional exposure across groups. However, unlike the experimental condition, learning in the control group did not incorporate project-based activities or student-centered inquiry processes. Instead, emphasis was placed on technical skill acquisition, teacher-directed practice, and performance repetition. This standardized instructional structure was maintained throughout the study to ensure that any observed differences in motivational outcomes could be attributed to the pedagogical approach rather than differences in instructional time or content exposure.

### Measures

Students’ motivation toward PE and sport participation was assessed using validated multidimensional measures grounded in SDT. Intrinsic motivation and identified regulation were measured using the Perceived Locus of Causality (PLOC) Scale for PE (Goudas et al., 1994). Each subscale comprised four items assessing students’ enjoyment and inherent interest in PE and their personal endorsement of its importance and benefits, respectively. Representative item content reflected participation because PE was enjoyable and personally valuable. Recent psychometric studies have further supported the factorial validity, construct validity, and reliability of the PLOC and its revised or culturally adapted versions among secondary-school PE students (Harbichová et al., 2025; Wolf et al., 2022). Behavioral intention was assessed using the five-item Intention to Be Physically Active (IPAS) Scale (Hein et al., 2004), which evaluates students’ intentions to remain physically active beyond compulsory PE. Recent research has continued to support its application in secondary-school PE settings (Fernández-Espínola et al., 2022; Fierro-Suero et al., 2023). In the present study, Cronbach’s alpha coefficients were *α* = 0.87 and 0.89 for intrinsic motivation, *α* = 0.84 and 0.86 for identified regulation, and *α* = 0.85 and 0.88 for behavioral intention at pretest and posttest, respectively. Together, these measures captured students’ autonomous motivational regulation and prospective intention to remain physically active. An overall motivation score was calculated as the mean of the intrinsic motivation, identified regulation, and behavioral intention subscale scores, with higher scores indicating stronger overall motivation toward PE and sport participation.

Basic psychological needs satisfaction was assessed using the Basic Psychological Needs in Physical Education (BPN-PE) Scale (Cagas and Hassandra, 2014; Langøy et al., 2024). The 12-item scale comprises three four-item dimensions assessing autonomy, competence, and relatedness. Autonomy reflects students’ perceptions of choice and volition during PE activities, competence reflects their perceived capability to perform and improve in PE-related tasks, and relatedness reflects their sense of interpersonal connection with classmates. Representative item content concerned having meaningful choices during PE, feeling capable of successfully performing challenging tasks, and experiencing positive relationships with classmates. Previous psychometric research has supported the three-factor structure, construct validity, and internal consistency of the BPN-PE among secondary-school students across different cultural contexts (Cagas and Hassandra, 2014; Langøy et al., 2024). In the present sample, Cronbach’s alpha coefficients were *α* = 0.85 and 0.88 for autonomy, *α* = 0.88 and 0.90 for competence, and *α* = 0.83 and 0.86 for relatedness at pretest and posttest, respectively. For the mediation analysis, overall psychological needs satisfaction was calculated as the mean of the autonomy, competence, and relatedness subscale scores, while the three dimensions were retained for construct-specific analyses.

Self-efficacy in PE was assessed using the nine-item student self-efficacy measure developed specifically for school-based PE by (Jackson et al., 2013). he measure assesses students’ confidence in successfully performing PE-related behaviors, including exerting effort, following instructional demands, persevering during challenging activities, learning and attempting new skills, and performing effectively in PE. Representative item content reflected confidence in learning skills taught in class, persisting with technically difficult activities, and attempting unfamiliar skills. Previous psychometric research has supported the factorial validity, reliability, and measurement invariance of the measure, while more recent evidence has reinforced the relevance of self-efficacy to students’ effort and enjoyment in PE (Hsu et al., 2022). In the present study, Cronbach’s alpha coefficients were *α* = 0.88 at pretest and *α* = 0.90 at posttest.

In the present study, PLOC, IPAS, and BPN-PE items were rated on a 5-point scale ranging from 1 (strongly disagree) to 5 (strongly agree), whereas self-efficacy items were rated from 1 (no confidence at all) to 5 (complete confidence). Higher scores indicated higher levels of the corresponding constructs, and subscale scores were calculated as the mean of the relevant item responses. Because the original instruments were developed in English, standardized translation and cultural-adaptation procedures were applied for use with Chinese secondary-school students. The questionnaires were independently translated into Chinese and back-translated into English by bilingual researchers, followed by expert review to ensure semantic, conceptual, and contextual equivalence. The Chinese versions were then pilot-tested with secondary-school students who were not included in the main sample to assess item clarity and comprehensibility, and minor wording adjustments were made where necessary without altering the intended meaning of the original items. The measures were selected based on their established use in school-based PE and their conceptual alignment with SDT for motivational regulation and psychological need satisfaction, and Social Cognitive Theory for self-efficacy. Evidence of measurement validity was considered not only from internal consistency estimates but also from the established factorial and construct validity of the original and culturally adapted instruments, together with expert evaluation and pilot testing of their content and contextual appropriateness for the present Chinese secondary-school population. Importantly, behavioral intention was recognized as conceptually distinct from motivational regulation; therefore, the overall motivation score was not intended to represent a unitary motivational-regulation construct within SDT. Rather, it was operationalized as a study-specific composite summary indicator capturing students’ broader motivational orientation toward PE and behavioral intention toward continued sport participation, combining autonomous motivational regulation represented by intrinsic motivation and identified regulation with prospective behavioral intention. To preserve the conceptual distinction among these constructs, intrinsic motivation, identified regulation, and behavioral intention were also analyzed and reported separately in the primary outcome analyses.

### Procedure

The study was conducted over a structured 12-week instructional period aligned with the school PE curriculum. The research procedure followed a three-phase quasi-experimental design, consisting of baseline assessment (pretest), intervention implementation, and post-intervention assessment (posttest). During the pretest phase, all participants completed standardized questionnaires measuring students’ motivation toward PE, basic psychological needs satisfaction, and self-efficacy. These baseline measurements were used to characterize pre-intervention levels and were subsequently incorporated as covariates in the mediation analysis. Following the pretest, the experimental group received the PBL-based PE intervention, while the control group continued with conventional teacher-centered instruction under regular PE teaching conditions. Both groups received instruction over the same duration and frequency to ensure consistency of exposure across conditions. After completion of the 12-week intervention period, the same measurement instruments were administered to both groups during the posttest phase to assess changes in motivational and psychological outcomes. Thus, psychological needs satisfaction, self-efficacy, and motivation were assessed at both baseline and immediately after the 12-week intervention; the post-intervention mediator and outcome measures were collected at the same assessment occasion. To ensure methodological rigor, the administration of pretest and posttest procedures was conducted under standardized conditions across all participating classes, minimizing external variation and ensuring consistency in data collection procedures.

### Data analysis

Data were analyzed using IBM SPSS Statistics (Version 22.0, IBM Corp., Armonk, NY, USA). Prior to analysis, the dataset was screened for missing values, outliers, and data-entry errors. Descriptive statistics were reported as means (M) and standard deviations (SD) for continuous variables and frequencies and percentages for categorical variables. Assumptions underlying the primary analyses were evaluated by inspecting standardized residuals, Q–Q plots, and residual-versus-fitted plots to assess approximate normality and homoscedasticity.

To evaluate intervention effects while accounting for both the repeated-measures structure and the clustering of students within intact classes, LMM were conducted separately for each study outcome. Group (PBL vs. control), time (pretest vs. posttest), and the group × time interaction were specified as fixed effects. Repeated measurements were nested within students, and students were nested within classes. Class was included as a random intercept to account for the non-independence of students within the eight intact classes (four PBL and four control classes). Class-level intraclass correlation coefficients (ICCs) were calculated from the variance components of the random-intercept mixed-effects models to quantify the proportion of outcome variance attributable to between-class differences. The group × time interaction constituted the primary test of intervention effectiveness, indicating whether the magnitude of pretest-to-posttest change differed between the PBL and control groups.

Following a significant group × time interaction, model-based planned pairwise contrasts were conducted to examine pretest-to-posttest changes within each group and between-group differences at posttest, with corresponding 95% confidence intervals (CIs). Bonferroni adjustment was applied to these comparisons to control for inflation of Type I error. Effect sizes for the group × time contrasts were expressed as Cohen’s *d*, calculated by standardizing the estimated interaction contrast using the residual SD from the corresponding mixed-effects model. Cohen’s *d* values of 0.20, 0.50, and 0.80 were interpreted as small, medium, and large effects, respectively.

To examine the hypothesized indirect pathways while accounting for the hierarchical structure of the data, a two-level parallel mediation analysis was conducted using Mplus (Version 8; Muthén & Muthén, Los Angeles, CA, USA). Students were specified at Level 1 and intact classes at Level 2. Because intervention condition was assigned at the class level, PBL versus control condition was modeled as a Level-2 predictor, whereas post-intervention psychological needs satisfaction, self-efficacy, and motivation were modeled as student-level variables. Random intercepts at the class level were estimated for the mediators and outcome to account for between-class variation and the non-independence of students within the same class.

Post-intervention psychological needs satisfaction and self-efficacy were entered simultaneously as parallel mediators, with post-intervention motivation specified as the outcome. Baseline motivation, psychological needs satisfaction, and self-efficacy were included as covariates to adjust for pre-intervention levels of the corresponding constructs. Models were estimated using maximum likelihood estimation with robust standard errors (MLR). Specific indirect effects were calculated as the products of the corresponding *a* and *b* paths, and 95% CIs were obtained from the multilevel model estimates. An indirect effect was considered statistically significant when its 95% CIs did not include zero.

Because only eight class-level clusters were available, the multilevel mediation analysis was considered a secondary exploratory analysis, and the magnitude and statistical precision of the indirect effects were interpreted cautiously. Because the mediators and outcome were assessed concurrently at posttest, the estimated indirect effects were interpreted as statistical indirect associations rather than definitive evidence of temporal or causal mediation. All statistical tests were two-tailed, with statistical significance set at *p* < 0.05.

## Results

### Preliminary analysis

Prior to the primary analyses, the dataset was screened for missing values, outliers, and data-entry errors. Descriptive statistics were calculated to summarize participant characteristics and baseline study outcomes. The assumptions of the LMM were evaluated through inspection of standardized residuals, Q–Q plots, and residual-versus-fitted plots. No substantial deviations from normality or homoscedasticity were observed, supporting the adequacy of the specified models. Baseline characteristics of the PBL and control groups are presented in Table 1. The groups showed similar descriptive characteristics in age, gender distribution, BMI, previous PE grade, and weekly physical activity. Because intervention effects were evaluated using mixed-effects models incorporating both pretest and posttest observations, baseline characteristics were summarized descriptively rather than subjected to significance testing. The study comprised eight intact classes, with four classes allocated to the PBL condition and four to the control condition. Class-level ICCs were estimated from the variance components of the random-intercept mixed-effects models to quantify the proportion of outcome variance attributable to differences between classes. The estimated ICC for overall motivation was 0.06, indicating that approximately 6% of the variance in overall motivation was attributable to between-class differences. Across the remaining study outcomes, class-level ICCs ranged from 0.04 to 0.09, indicating modest within-class clustering and supporting the use of mixed-effects models that accounted for the hierarchical structure of the data. Figure 2 illustrates participant recruitment, class-level allocation, intervention procedures, post-intervention assessment, and analysis.

**Table 1:** Participant characteristics.

Variable	PBL group (*n* = 60)	Control group (*n* = 60)
Intact classes, *n*	4	4
Age (years)	15.62 ± 1.12	15.58 ± 1.09
Gender (Male/Female)	32/28	31/29
BMI (kg/m^2^)	21.34 ± 2.11	21.41 ± 2.05
Previous PE grade	78.6 ± 6.8	79.1 ± 6.5
Weekly physical activity (hours)	3.12 ± 1.21	3.08 ± 1.18

**Figure 2:**
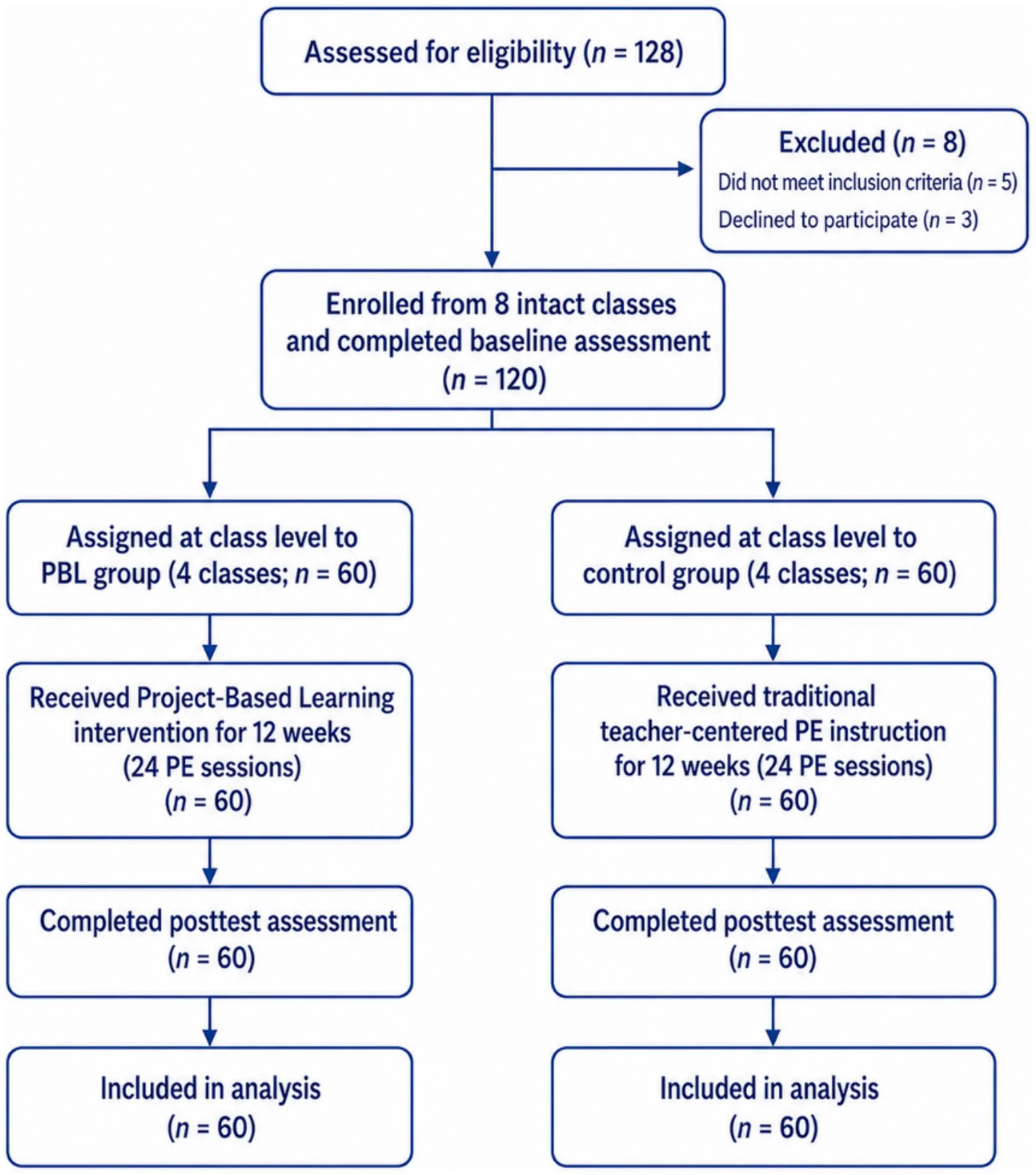
Flow diagram of participant recruitment, allocation, intervention, and data analysis.

### Descriptive statistics

Descriptive statistics for all study outcomes at pretest and posttest are presented in Table 2. Baseline scores were broadly comparable between the PBL and control groups across motivational, psychological needs, and self-efficacy outcomes. Descriptively, the PBL group showed larger pretest-to-posttest increases across all measured constructs, whereas comparatively smaller changes were observed in the control group. The largest increases in the PBL group were observed for competence, intrinsic motivation, autonomy, and overall psychological needs satisfaction. These descriptive patterns were subsequently evaluated using LMM, with the group × time interaction serving as the primary inferential test of differential change between the PBL and control groups.

**Table 2:** Pretest–posttest descriptive statistics for study outcomes by group.

Outcome	PBL (*n* = 60), Pretest (*M* ± SD)	PBL (*n* = 60), Posttest (*M* ± SD)	Control (*n* = 60), Pretest (*M* ± SD)	Control (*n* = 60), Posttest (*M* ± SD)
Overall motivation	3.12 ± 0.42	4.21 ± 0.38	3.10 ± 0.40	3.42 ± 0.45
Intrinsic motivation	3.05 ± 0.44	4.30 ± 0.36	3.08 ± 0.41	3.50 ± 0.43
Identified regulation	3.14 ± 0.43	4.10 ± 0.40	3.11 ± 0.42	3.39 ± 0.44
Behavioral intention	3.17 ± 0.41	4.23 ± 0.39	3.11 ± 0.40	3.37 ± 0.42
Psychological needs satisfaction	3.18 ± 0.39	4.38 ± 0.37	3.16 ± 0.40	3.60 ± 0.42
Autonomy	3.15 ± 0.40	4.38 ± 0.38	3.15 ± 0.41	3.60 ± 0.43
Competence	3.18 ± 0.38	4.46 ± 0.35	3.18 ± 0.39	3.62 ± 0.40
Relatedness	3.21 ± 0.42	4.30 ± 0.40	3.15 ± 0.41	3.58 ± 0.43
Self-Efficacy	3.18 ± 0.45	4.32 ± 0.39	3.20 ± 0.43	3.58 ± 0.44

### Effects of project-based learning on motivation

LMM analyses revealed significant group × time interactions for overall motivation and all motivational subdimensions, indicating that pretest-to-posttest changes differed significantly between the PBL and control groups. As shown in Table 3, a significant group × time interaction was observed for overall motivation, *F*(1, 6) = 41.17, *p* < 0.001, *d* = 1.60, indicating a substantially greater improvement in the PBL group than in the control group. Significant group × time interactions were also observed for intrinsic motivation, *F*(1, 6) = 47.84, *p* < 0.001, *d* = 1.67; identified regulation, *F*(1, 6) = 36.21, *p* < 0.001, *d* = 1.35; and behavioral intention, *F*(1, 6) = 44.44, *p* < 0.001, *d* = 1.48. Collectively, these findings indicate that PBL was associated with substantially greater improvements in overall motivation, autonomous motivational regulation, and students’ prospective intention to remain physically active compared with traditional PE instruction.

**Table 3:** LMM results for group × time effects across study outcomes.

Outcome	Estimated group × time effect	95% CI	*F* (1, 6)	*p*-value	Cohen’s d	Magnitude
Overall motivation	0.77	0.48–1.06	41.17	<0.001	1.60	Large
Intrinsic motivation	0.83	0.54–1.12	47.84	<0.001	1.67	Large
Identified regulation	0.68	0.40–0.96	36.21	<0.001	1.35	Large
Behavioral intention	0.80	0.51–1.09	44.44	<0.001	1.48	Large
Psychological needs satisfaction	0.76	0.49–1.03	47.74	<0.001	1.68	Large
Autonomy	0.78	0.52–1.04	55.18	<0.001	1.72	Large
Competence	0.84	0.58–1.10	64.00	<0.001	1.80	Large
Relatedness	0.66	0.42–0.90	43.56	<0.001	1.62	Large
Self-Efficacy	0.76	0.50–1.02	52.39	<0.001	1.70	Large

### Effects on basic psychological needs satisfaction

LMM analyses revealed significant group × time interactions for overall psychological needs satisfaction and each of its three dimensions, indicating greater pretest-to-posttest improvements in the PBL group than in the control group. A significant interaction was observed for autonomy, *F*(1, 6) = 55.18, *p* < 0.001, *d* = 1.72, indicating a substantially greater increase in students’ perceived choice and volition in the PBL group. Competence showed the largest effect, *F*(1, 6) = 64.00, *p* < 0.001, *d* = 1.80, reflecting a substantially greater increase in students’ perceived capability to perform and master PE-related tasks in the PBL group. Relatedness also demonstrated a significant group × time interaction, *F*(1, 6) = 43.56, *p* < 0.001, *d* = 1.62, reflecting greater improvement in students’ perceived interpersonal connection within the PE environment. The composite psychological needs satisfaction score similarly showed a significant group × time interaction, *F*(1, 6) = 47.74, *p* < 0.001, *d* = 1.68. Collectively, these findings indicate that PBL was associated with substantially greater improvements across autonomy, competence, and relatedness than traditional PE instruction.

### Effects on self-efficacy in physical education

LMM analysis revealed a significant group × time interaction for self-efficacy, *F*(1, 6) = 52.39, *p* < 0.001, *d* = 1.70, indicating that changes in self-efficacy from pretest to posttest differed significantly between the PBL and control groups. As shown in Table 2, self-efficacy increased from 3.18 ± 0.45 to 4.32 ± 0.39 in the PBL group, whereas the control group showed a smaller increase from 3.20 ± 0.43 to 3.58 ± 0.44. The large effect size indicated a substantial intervention-related difference in the magnitude of change. These findings indicate that students in the PBL group exhibited substantially greater improvements in perceived self-efficacy than those in the traditional PE instruction group.

### Planned pairwise comparisons

Following the significant group × time interactions, Bonferroni-adjusted planned pairwise comparisons were conducted to further characterize the pattern of change within each group and the between-group differences at posttest. Within the PBL group, significant pretest-to-posttest improvements were observed across all motivational and psychological outcomes, including overall motivation, intrinsic motivation, identified regulation, behavioral intention, psychological needs satisfaction, autonomy, competence, relatedness, and self-efficacy (all adjusted *p* < 0.001). The largest descriptive increases were observed for competence (3.18 ± 0.38 to 4.46 ± 0.35), intrinsic motivation (3.05 ± 0.44 to 4.30 ± 0.36), autonomy (3.15 ± 0.40 to 4.38 ± 0.38), and overall psychological needs satisfaction (3.18 ± 0.39 to 4.38 ± 0.37). The control group exhibited substantially smaller changes over the same period. Significant but comparatively modest improvements were observed for intrinsic motivation, psychological needs satisfaction, autonomy, competence, relatedness, and self-efficacy (adjusted *p* < 0.05), whereas changes in overall motivation, identified regulation, and behavioral intention did not remain statistically significant after Bonferroni adjustment (adjusted *p* > 0.05).

At posttest, the PBL group demonstrated significantly higher scores than the control group across all study outcomes. The adjusted between-group difference was 0.79 for overall motivation [95% CI (0.51, 1.07), adjusted *p* < 0.001], 0.80 for intrinsic motivation [95% CI (0.52, 1.08), adjusted *p* < 0.001], 0.71 for identified regulation [95% CI (0.43, 0.99), adjusted *p* < 0.001], and 0.86 for behavioral intention [95% CI (0.58, 1.14), adjusted *p* < 0.001]. Similarly, posttest differences favored PBL for overall psychological needs satisfaction [mean difference = 0.78, 95% CI (0.51, 1.05)], autonomy [0.78, 95% CI (0.51, 1.05)], competence [0.84, 95% CI (0.58, 1.10)], relatedness [0.72, 95% CI (0.46, 0.98)], and self-efficacy [0.74, 95% CI (0.47, 1.01)]; all adjusted *p* < 0.001. Taken together, the planned contrasts indicated that the significant group × time interactions were characterized by substantially greater pretest-to-posttest improvements in the PBL group and consistently higher outcome scores in the PBL group at the end of the 12-week intervention.

### Associations of psychological needs satisfaction and self-efficacy with motivation

Within the baseline-adjusted parallel mediation model, both psychological needs satisfaction and self-efficacy were positively associated with students’ post-intervention motivation. After controlling for intervention condition and corresponding baseline scores, psychological needs satisfaction was positively associated with motivation [*B* = 0.33, SE = 0.07, 95% CI (0.19, 0.47), *p* < 0.001]. Similarly, self-efficacy showed a significant positive association with post-intervention motivation [*B* = 0.25, SE = 0.06, 95% CI (0.13, 0.37), *p* < 0.001]. These findings indicate that students reporting greater satisfaction of autonomy, competence, and relatedness, as well as stronger confidence in their ability to perform PE-related tasks, tended to demonstrate higher levels of motivation after the intervention. The results therefore support the hypothesized positive associations of psychological needs satisfaction and self-efficacy with motivational outcomes.

### Multilevel analysis of indirect associations

A secondary two-level parallel mediation analysis was conducted to examine whether psychological needs satisfaction and self-efficacy were indirectly associated with the relationship between PBL condition and post-intervention motivation while accounting for the clustering of students within intact classes. Students were modeled at Level 1 and classes at Level 2, with class-level random intercepts included for the mediators and outcome. Baseline motivation, psychological needs satisfaction, and self-efficacy were included as covariates. The multilevel analysis indicated a significant indirect association of PBL with post-intervention motivation through psychological needs satisfaction [*B* = 0.22, SE = 0.09, 95% CI (0.05, 0.39)] and through self-efficacy [*B* = 0.16, SE = 0.08, 95% CI (0.02, 0.30)]. The total indirect effect across both mediators was also significant [*B* = 0.38, SE = 0.12, 95% CI (0.14, 0.62)]. The direct association between PBL condition and post-intervention motivation remained significant after accounting for both mediators and baseline covariates [*B* = 0.30, SE = 0.12, 95% CI (0.06, 0.54)], a pattern consistent with partial mediation. The total association between PBL condition and post-intervention motivation was *B* = 0.68 [SE = 0.13, 95% CI (0.42, 0.94)].

Figure 3 illustrates the estimated direct and indirect associations linking PBL condition with post-intervention motivation through psychological needs satisfaction and self-efficacy within the multilevel framework. Collectively, these findings suggest that psychological needs satisfaction and self-efficacy may represent complementary psychological pathways associated with the greater motivational improvement observed in the PBL group. However, because the mediators and outcome were measured concurrently at posttest, temporal precedence cannot be established, and these indirect effects should not be interpreted as definitive evidence of causal mediation (see Table 4).

**Figure 3:**
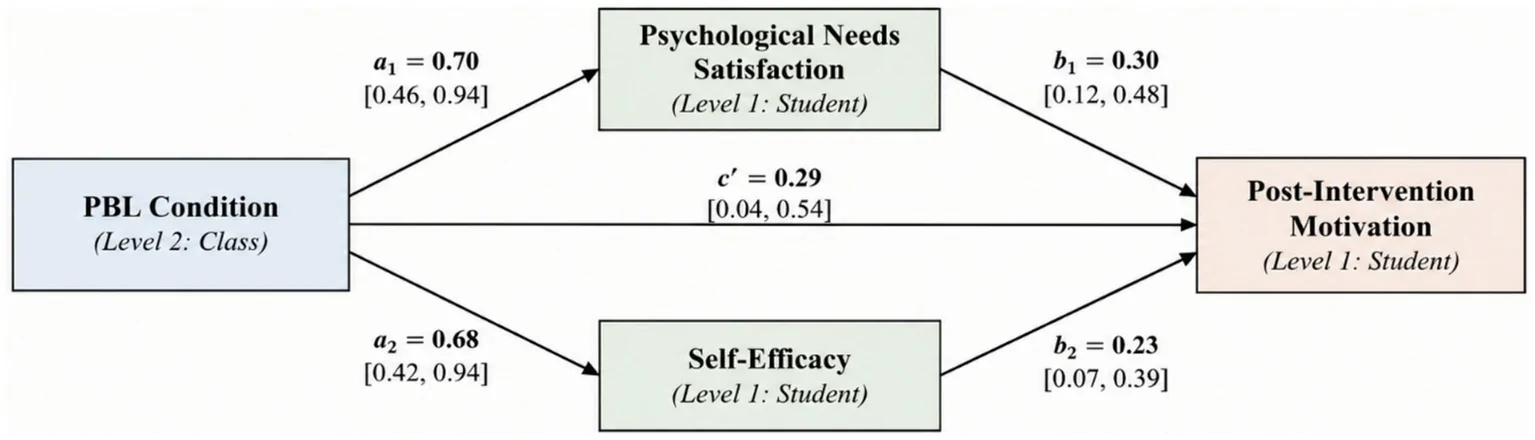
Two-level model of indirect associations linking PBL with post-intervention motivation through psychological needs satisfaction and self-efficacy. Arrows represent statistical associations specified in the multilevel model and should not be interpreted as evidence of causal direction.

**Table 4:** Two-level parallel mediation analysis accounting for class-level clustering.

Path/effect	*B*	SE	95% CI	*p*-value/inference
PBL → psychological needs satisfaction (*a₁*)	0.70	0.12	[0.46, 0.94]	< 0.001
PBL → self-efficacy (*a₂*)	0.68	0.13	[0.42, 0.94]	< 0.001
Psychological needs satisfaction → motivation (*b₁*)	0.30	0.09	[0.12, 0.48]	0.001
Self-efficacy → motivation (*b₂*)	0.23	0.08	[0.07, 0.39]	0.005
PBL → motivation, direct effect (*c′*)	0.29	0.13	[0.04, 0.54]	0.024
PBL → psychological needs satisfaction → motivation	0.21	0.09	[0.04, 0.38]	Significant indirect effect
PBL → self-efficacy → motivation	0.16	0.07	[0.02, 0.30]	Significant indirect effect
Total indirect effect	0.37	0.11	[0.15, 0.59]	Significant
Total effect (*c*)	0.66	0.15	[0.37, 0.95]	< 0.001

## Discussion

This study provides empirical and theoretical evidence that PBL is associated with enhanced students’ motivation toward PE and school-based sport participation. The findings indicate that students exposed to PBL exhibited significantly higher levels of intrinsic motivation, psychological needs satisfaction, self-efficacy, and behavioral intention compared to those receiving traditional instruction (Moritz et al., 2000). These results not only support all proposed hypotheses but also extend current knowledge by providing insight into the potential psychological and pedagogical pathways linking instructional design with motivational outcomes in PE contexts.

A key contribution of this study lies in providing empirical support for key assumptions of SDT within a real-world school sport setting. The results show that participation in the PBL condition was associated with significantly greater improvements in autonomy, competence, and relatedness, which are the core determinants of self-determined motivation (Peng et al., 2025). Among these, competence exhibited the largest standardized effect, followed closely by autonomy, suggesting that PBL may be particularly effective in strengthening students’ perceptions of mastery and volition in PE (Ferrero et al., 2021a,b). This finding is consistent with SDT literature emphasizing that autonomy-supportive environments are critical for internalizing motivation, especially in adolescent populations where engagement in physical activity is highly sensitive to perceived control and relevance of learning tasks.

The significant improvement in perceived competence further highlights the importance of mastery-based learning experiences in PE. Unlike traditional drill-based instruction, PBL provides iterative opportunities for students to engage in meaningful sport-related tasks, receive feedback, and refine performance over time (Azizan et al., 2013; English, 2018). This experiential cycle appears to strengthen students’ perceptions of effectiveness in physical tasks, which is a key predictor of sustained participation in physical activity (Cach and Carrasco-venturelli, 2023). In this sense, the findings reinforce the argument that competence development in PE is not solely dependent on technical repetition but is significantly enhanced through authentic, problem-solving-based learning environments (Van Den Berg et al., 2021; Hind et al., 2025). Relatedness was also significantly improved in the PBL condition, underscoring the social value of collaborative learning in PE (Taylor et al., 2008; Ullrich-french et al., 2013). The group-based structure of PBL fosters peer interaction, shared responsibility, and collective achievement, which are essential components for building a supportive motivational climate. This is particularly relevant in school sport contexts, where social belonging often determines long-term engagement. The present findings suggest that PBL may serve as a social catalyst that strengthens interpersonal connections, thereby enhancing motivational resilience in PE participation.

From a social cognitive perspective, the significant increase in self-efficacy provides further insight into the motivational processes associated with PBL (Guo et al., 2020; Langøy et al., 2024). Students who participated in PBL demonstrated greater confidence in their ability to perform physical and sport-related tasks. These improvements may be related to repeated mastery experiences, peer modeling, and constructive feedback embedded within project-based activities (Dishman et al., 2005). Importantly, the multilevel analysis identified a significant indirect association through self-efficacy while accounting for the clustering of students within classes, suggesting that beliefs about capability may represent one potential pathway linking PBL with motivation and behavioral intentions toward sport participation (Buayai et al., 2026; Shen et al., 2009).

The analysis of indirect associations further contributes to the theoretical interpretation of this study by identifying significant indirect associations through psychological needs satisfaction and self-efficacy while accounting for the hierarchical structure of students nested within classes (Aelterman et al., 2018; Haerens et al., 2015). These findings are consistent with a potential dual-pathway explanation of how pedagogical design may relate to motivational processes. Specifically, greater psychological needs satisfaction and self-efficacy in the PBL group were associated with higher post-intervention motivation, consistent with the possibility that these constructs represent complementary psychological pathways related to students’ motivational responses (Huéscar et al., 2020; Lochbaum et al., 2023). This integrated theoretical perspective extends current literature by linking constructivist pedagogy with motivational psychology within a unified explanatory framework.

From a broader educational perspective, these findings suggest that traditional teacher-centered PE models may be less effective in supporting students’ motivation and intentions toward continued sport participation (Hein, 2016; Sheeran et al., 2020). In contrast, PBL represents a transformative pedagogical approach that aligns instructional design with psychological principles of motivation. By embedding learning within authentic sport-related projects, PBL bridges the gap between classroom learning and real-world physical activity engagement, thereby increasing the relevance and meaningfulness of PE for students (Lee and Patall, 2024).

Importantly, this study contributes a novel empirical perspective by situating PBL specifically within the context of school-based PE and sport participation, an area that remains underexplored in existing literature. While previous research has predominantly focused on cognitive or academic outcomes of PBL in general education, the present findings extend its applicability to motivational and behavioral outcomes in sport settings. This expansion is theoretically significant because it indicates that pedagogical innovation may influence not only learning-related outcomes but also motivational processes and intentions toward future sport engagement.

The magnitude of the observed effects warrants cautious interpretation. Cohen’s *d* values ranged from 1.35 to 1.80, which are unusually large for educational and psychological interventions. These effects may partly reflect the strong contrast between the structured, student-centered PBL condition and the predominantly teacher-centered control condition. However, the intact-class quasi-experimental design, potential teacher- and classroom-level influences, reliance on self-report Likert measures, and relatively low within-group variability may also have contributed to the large standardized effects. In addition, the novelty and increased attention associated with participation in the PBL condition may have contributed to a Hawthorne effect, whereby students responded more positively because they were aware of participating in a distinctive instructional intervention. Because all motivational and psychological outcomes were assessed using self-report measures, social desirability bias may also have inflated students’ reported improvements. Furthermore, although each teacher taught both PBL and control classes, teachers were necessarily aware of the instructional condition, creating the possibility of teacher expectancy effects through subtle differences in enthusiasm, feedback, encouragement, or interaction with students across conditions. Consequently, the unusually large effect sizes should not be attributed solely to the pedagogical effect of PBL, but should be interpreted as potentially reflecting both intervention-related changes and contextual or measurement-related influences. Accordingly, these findings should be interpreted within the specific methodological, instructional, and measurement context of the study. Replication using larger cluster-randomized designs, multiple schools and teachers, objective behavioral outcomes, and longer-term follow-up is needed to confirm their robustness and generalizability.

Despite these contributions, several limitations should be acknowledged. The quasi-experimental design, while appropriate for educational settings, limits the ability to infer causal relationships with absolute certainty. Although instructional procedures were standardized and each teacher contributed to both conditions, potential teacher- and school-level influences cannot be completely excluded; moreover, the relatively small number of participating classes and the achieved sample size slightly below the *a priori* target warrant cautious interpretation. Although separate instructional protocols, condition-specific lesson plans, and fidelity monitoring were used to minimize cross-contamination, residual spillover between the PBL and control conditions cannot be completely excluded because the same teachers taught both instructional conditions. In addition, the initial G*Power calculation was based on individual-level assumptions and did not incorporate the hierarchical structure of students nested within classes or the corresponding design effect associated with the class-level ICC; therefore, the effective statistical power may have been lower than suggested by the original estimate. Additionally, the reliance on self-reported outcomes may have introduced social desirability and response biases, while awareness of the intervention condition among students and teachers may have contributed to Hawthorne and teacher expectancy effects. Although the mediation analysis explicitly accounted for class-level clustering using a multilevel framework, the relatively small number of class-level clusters (eight classes) may limit the precision of between-class estimates and therefore warrants cautious interpretation of the indirect effects. The proposed theoretical framework was examined using multilevel parallel mediation rather than a full structural equation model; therefore, the relationships among all constructs were not tested simultaneously within a comprehensive structural model. Future studies with larger samples and more clusters should employ SEM or multilevel SEM to provide a more comprehensive validation of the framework. External validity is also limited by the specific educational context, the exclusive inclusion of secondary school students, and the relatively short 12-week intervention period. Because no longitudinal follow-up was conducted, the present findings cannot establish whether the observed motivational and psychological improvements persist over time or translate into sustained sport participation. Because behavioral intention was assessed rather than actual sport participation, the study cannot determine whether stronger intentions translated into objectively verified physical activity or sport behavior. Future research should therefore incorporate longer-term follow-up assessments and objective behavioral measures, such as accelerometer-assessed physical activity and school sports club attendance records, to determine whether improvements in behavioral intention translate into actual and sustained participation. Moreover, replication across diverse cultural and educational contexts is necessary to confirm the generalizability of the findings.

In conclusion, this study provides evidence that PBL is associated with meaningful improvements in students’ motivation toward PE and sport participation. By integrating motivational theory with constructivist pedagogy, the findings highlight potential psychological pathways through which educational design may be associated with motivational outcomes. This contributes to the advancement of sport pedagogy by positioning PBL as a promising instructional approach for enhancing motivational and psychological factors that may support continued engagement in physical activity among school-aged students.

## Conclusion

This study examined the relationship between PBL and students’ motivation toward PE and school-based sport participation within an integrated theoretical framework combining SDT, constructivist learning theory, and social cognitive theory. The findings provide empirical evidence that students in the PBL condition showed greater improvements in motivational and psychological outcomes than those receiving traditional teacher-centered instruction.

Specifically, students exposed to PBL demonstrated higher levels of intrinsic motivation, stronger behavioral intention toward sport participation, greater satisfaction of psychological needs (autonomy, competence, and relatedness), and higher self-efficacy in PE. These results support the potential of PBL to enhance both the level and quality of students’ motivation by creating conditions associated with more self-determined engagement.

The results also provide support for the proposed conceptual framework, with significant direct and indirect associations observed between PBL condition and post-intervention motivation. Psychological needs satisfaction and self-efficacy may represent complementary psychological pathways linking PBL with motivational outcomes. Given that the mediators and outcome were measured concurrently at posttest, however, these indirect associations should not be interpreted as definitive evidence of causal mediation. This integrated explanation strengthens the theoretical contribution of the study by linking instructional innovation with psychological processes in PE.

From a practical perspective, the findings suggest that incorporating PBL into PE curricula may support more active and self-determined student engagement. By creating autonomy-supportive, competence-enhancing, and socially collaborative learning environments, PBL offers a pedagogically meaningful approach to supporting students’ motivation and intentions toward continued sport participation.

In conclusion, PBL represents a promising and theoretically grounded pedagogical approach in PE that is associated with meaningful improvements in motivational and psychological outcomes. Future research should investigate whether these improvements persist over time and translate into actual sport participation behavior, and examine the applicability of PBL across diverse educational systems and cultural contexts.

## Data Availability

The original contributions presented in the study are included in the article/supplementary material, further inquiries can be directed to the corresponding author.
